# Diagnostic Challenges in Epithelioid Pleural Mesothelioma: Case Series with Support from Electron Microscopy

**DOI:** 10.3390/diagnostics11050841

**Published:** 2021-05-07

**Authors:** Francesco Fortarezza, Mila Della Barbera, Federica Pezzuto, Francesca Lunardi, Eleonora Faccioli, Giulia Pasello, Federico Rea, Stefania Rizzo, Fiorella Calabrese

**Affiliations:** 1 Department of Cardiac, Thoracic, Vascular Sciences and Public Health, Cardiovascular Pathology Unit, University of Padova, 35128 Padova, Italy; francescofortarezza.md@gmail.com (F.F.); mila.dellabarbera@unipd.it (M.D.B.); federica.pezzuto@phd.unipd.it (F.P.); francesca.lunardi@unipd.it (F.L.); s.rizzo@unipd.it (S.R.); 2 Department of Cardiac, Thoracic, Vascular Sciences and Public Health, Thoracic Surgery Unit, University of Padova, 35128 Padova, Italy; faccioli.eleonora@gmail.com (E.F.); federico.rea@unipd.it (F.R.); 3 Medical Oncology 2, Istituto Oncologico Veneto IRCCS, 35128 Padova, Italy; giulia.pasello@iov.veneto.it; 4 Department of Surgery, Oncology and Gastroenterology, University of Padova, 35128 Padova, Italy

**Keywords:** mesothelioma, histology, immunohistochemistry, transmission electron microscopy

## Abstract

The histological diagnosis of pleural epithelioid mesothelioma can be difficult in the case of rare variants or in the definition of neoplasm origin in patients with previous or concomitant tumours. Currently, several immunohistochemical reactions are available in the surgical pathologist’s armamentarium that allow us to obtain a more sensitive and specific diagnosis of malignant pleural mesothelioma. However, in some cases, the final interpretation remains inconclusive. Historically, ultrastructural examination has represented a useful tool for the definition of the mesothelial nature of neoplastic cells due to their peculiar morphological characteristics. The recent international guidelines for pathological diagnosis of pleural mesothelioma suggest the use of electron microscopy when the immunohistochemical reactions are equivocal or when further support of a diagnosis of mesothelioma is needed. This paper presents three cases of pleural epithelioid mesothelioma whose diagnoses were finally supported by ultrastructural examination.

## 1. Introduction

Pleural malignant mesothelioma is a rare and fatal neoplasm that, in most cases, affects elderly patients who have had occupational exposure to asbestos [1]. The most common variant of the neoplasm is diffuse malignant mesothelioma, which is currently classified in epithelioid, biphasic, and sarcomatoid histotypes with different prognostic values [2]. The definite diagnosis of mesothelioma requires histopathological examination of pleural tissue, in most cases obtained from laparoscopic or thoracoscopic procedures [1]. Histological diagnosis is based on recognition of the mesothelial nature of neoplastic cells and on the invasive aspects of the subpleural fat. In the case of adequate sampling of the pleural tissues [3], assessment of the mesothelial origin may present diagnostic challenges for pathologists, especially in the case of rare histological variants or in the differential diagnosis of pleural metastasis. Immunohistochemical reactions (such as Calretinin, D2-40, and WT1) play a fundamental role in defining the mesothelial origin of the tumour [4]. Moreover, in the pathologist’s armamentarium, several other antibodies are currently used, the most important of which are BAP1 and MTAP. The lack of nuclear expression of both markers represents a molecular epiphenomenon of these crucial tumour suppressor genes whose alterations are responsible for mesothelioma carcinogenesis. However, none of these markers are 100% specific; indeed, the international guidelines recommend that at least two mesothelial and two carcinoma markers should be included in any immunohistochemical panel [4].

Another now-discussed tool to detect the mesothelial origin of the neoplastic cell is represented by transmission electron microscopy (TEM) [5]. The ultrastructural examination of the cells may highlight the findings of mesothelial origin, such as elongated microvilli, desmosome junctions, or intracytoplasmic tonofilaments [6].

The introduction of immunohistochemistry and molecular pathology has progressively restricted the use of TEM for diagnostic purposes, especially in the oncologic field. However, TEM still is a useful adjuvant tool in challenging diagnoses, as in cases such as that of epithelioid pleural mesothelioma that are described in this paper. In the discussion section, we provide a short review of the limited literature currently available and comment on some important messages.

## 2. Case Description

### 2.1. Case 1: An Unusual Morphological Finding

**Figure 1:**
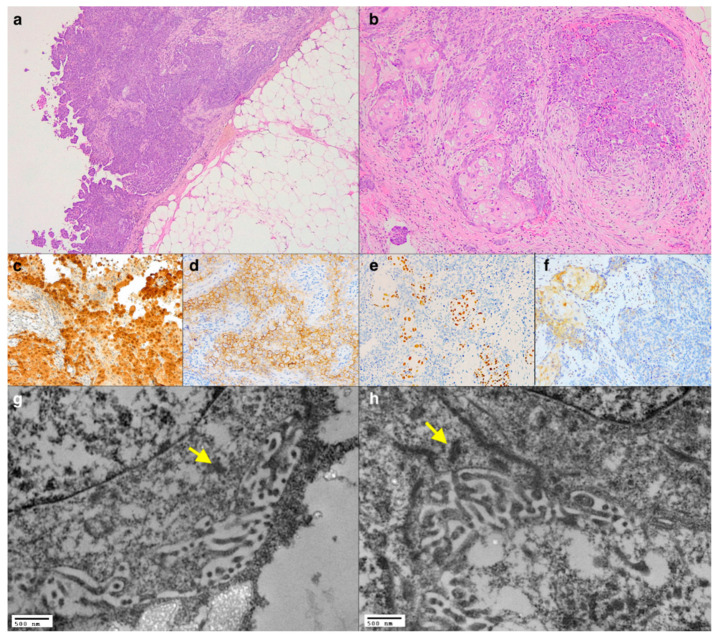
Panoramic view of the pleural tumour showing a prevalent solid pattern with papillary fronds (**a**, haematoxylin and eosin stain, original magnification ×50), and several foci of squamous differentiation were evident at higher magnification (**b**, haematoxylin and eosin stain, original magnification ×100). Immunohistochemistry showing the positivity of Calretinin (**c**, immunoperoxidase staining, original magnification ×200), D2-40 (**d**, immunoperoxidase staining, original magnification ×400), positivity of p40 in the squamous cells (**e**, immunoperoxidase staining, original magnification ×200), and loss of nuclear expression of BAP1 in both the neoplastic components (**f**, immunoperoxidase staining, original magnification ×200). TEM showing the presence of microvilli and tight junctions (yellow arrows) (**g**,**h**, original magnification ×20,000).

A 72-year-old, non-smoker male with occupational exposure to asbestos presented with cough and chest pain. History was negative for previous or concomitant malignancies. Computed tomography (CT) showed right pleural effusion with pleural thickening that was biopsied. At histology, it was observed a neoplastic proliferation morphologically compatible with mesothelioma with several foci of squamous differentiation (Figure 1a,b). Immunohistochemical reactions were positive for markers of mesothelial origin (Figure 1c,d), while in the squamous component, the neoplastic cells strongly expressed p40 (Figure 1e). Interestingly, both components showed the nuclear loss of BAP1 expression (Figure 1f). Given the unusualness of these immunomorphological findings, ultrastructural examination was carried out on formalin-fixed paraffin-embedded (FFPE) sections (Figure 1g,h). In all cases, we followed the same protocol for processing specimens from paraffin blocks [7]. The surface of most of the neoplastic cells showed the characteristic branched microvilli, thus corroborating the final diagnosis of epithelioid mesothelioma with areas of squamous metaplasia.

### 2.2. Case 2: A Single Intrathoracic Nodule

**Figure 2:**
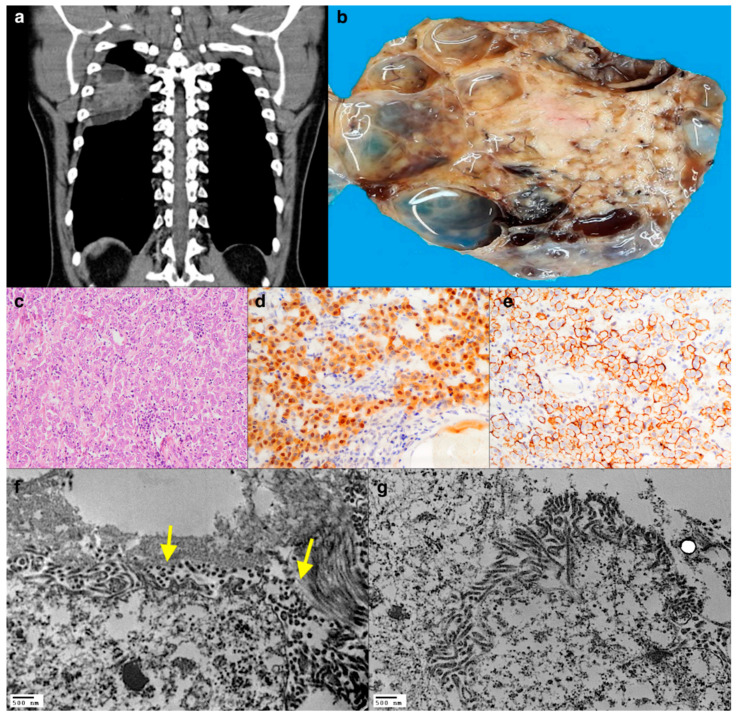
CT scan showing an intrathoracic mass with peripheric cystic changes (**a**), as visible at the cut surface of the gross specimen (**b**). At histology, the tumour comprised epithelioid cells with eosinophilic cytoplasm and brisk lymphocytic infiltrate (**c**, haematoxylin and eosin stain, original magnification ×200) that were immunoreactive for Calretinin (**d**, immunoperoxidase staining, original magnification ×200) and D2-40 (**e**, immunoperoxidase staining, original magnification ×200). TEM showing the presence of numerous microvilli in direct contact with collagen bundles (yellow arrows). Although some morphological details are affected by a non-optimal conservation, the structures useful for the diagnostic definition of mesothelioma appear to be well preserved (**f**,**g**, original magnification ×12,000, ×10,000).

An unexposed, non-smoking, 41-year-old man was admitted to the emergency room due to an accidental fall and tibial fracture. Pre-surgical radiological tests showed a large posterior, pleural-based, and well-defined nodular lesion in the right hemithorax. The patient underwent a complete surgical resection of the tumour. The surgical inspection of the pleural cavity was negative for other nodules with no pleural thickening and no pleural effusion. The mass showed a solid central portion and peripheral cystic and necrotic-haemorrhagic areas (Figure 2a,b). The tumour was composed of large cells with abundant eosinophilic cytoplasm (Figure 2c), positive for all mesothelial markers (Figure 2d,e). At electron microscopy examination, all the neoplastic cells showed the presence of elongated microvilli in contact with collagen bundles without the interposition of a basement membrane (Figure 2f,g). The final histological diagnosis of localized malignant mesothelioma (LMM) with deciduous changes was made.

### 2.3. Case 3: Primitive or Metastasis?

**Figure 3:**
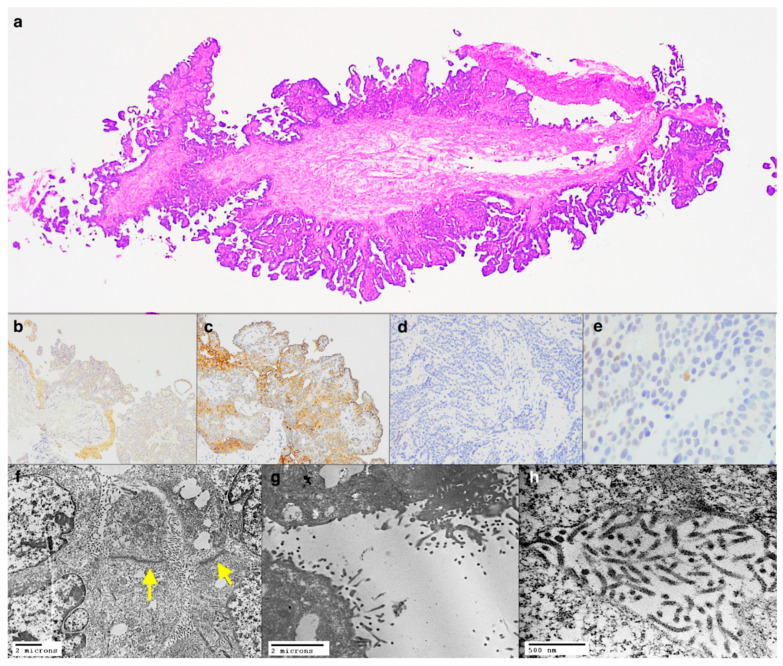
Panoramic view of the pleural biopsy showing a papillary proliferation (**a**, haematoxylin and eosin stain, original magnification ×25). Immunohistochemistry showing weak positivity for Calretin (**b**, immunoperoxidase staining, original magnification ×100), D2-40 (**c**, immunoperoxidase staining, original magnification ×200), loss of nuclear expression of BAP1 (**d**, immunoperoxidase staining, original magnification ×200), and rare cells positive for estrogen receptor (**e**, immunoperoxidase staining, original magnification ×400). TEM showing tight junctions (yellow arrows) and branched and elongated microvilli (**f**–**h**, original magnification ×3500, ×7000, ×30,000).

An unexposed, 69-year-old woman presented with a right pleural effusion. She had a history of right breast cancer treated with a quadrantectomy with adjuvant chemo-radiotherapy followed then by right mastectomy for locoregional recurrence. Medical thoracoscopy showed significant pleural effusion and multiple nodules. At histology, a diffuse epithelioid growth pattern with papillary structures was seen in the biopsy fragments (Figure 3a). Immunohistochemistry showed weak positivity for calretinin (Figure 3b), D2-40 (Figure 3c), and GATA3 while showing negativity for TTF1, GCDFP-15, BAP1 (Figure 3d), HER2, and progesterone receptor. Estrogen receptors were weakly positive in only 5% of neoplastic cells (Figure 3e). Therefore, the immunohistochemical findings were not decisive in differentiating a pleural localization of the breast cancer versus a primary tumour of the pleura. Ultrastructural analyses from FFPE sections showed most of the neoplastic cells with elongated, branched microvilli without glycocalyx and with tight junctions (Figure 3f–h). Based on these findings, the diagnosis was malignant epithelioid mesothelioma of the pleura. The patient underwent a pleurectomy after adjuvant chemotherapy. The histological analysis of the surgical specimen further confirmed the diagnosis of mesothelioma rather easily through a better yield of immunohistochemical analyses.

## 3. Discussion

The recent guidelines for pathological diagnosis of malignant mesothelioma [4] report that “occasionally the electron microscopy is useful in establishing the correct diagnosis when the immunohistochemical results are equivocal or when further support of a diagnosis of either malignant mesothelioma or serous carcinoma is needed”. The ultrastructural features of epithelioid mesothelioma cells are well known. The main distinguishing finding is the presence of abundant, undulating, long, and often bifurcating microvilli. These lack filamentous cores and surface glycocalyx; they measure approximately 0.1 μm in diameter and up to 3 μm in length. Other additional characteristics of the mesothelial nature are the presence of bundles of intracytoplasmic tonofilaments, present in the perinuclear areas or in relation to the desmosomal junctions which in mesothelial cells can be longer than a micrometer, the so-called giant desmosomes [6]. In the past, due to these peculiar characteristics, electron microscopy was considered the gold standard for the diagnosis of epithelioid mesothelioma. Currently, it is being largely replaced by immunohistochemistry, allowing for an easier diagnosis even if there is no complete sensitivity and specificity standardization on the clones to use and on their positivity cut-offs. Some authors still suggest the use of TEM to establish the mesothelial origin of the tumour rather than the recurrence of a note neoplasm [8] or to better characterize unusual and tricky variants, such as those in which mesothelioma shows lepidic growth in the lung parenchyma [9,10].

The three cases described represent a diagnostic challenge in defining pleural neoplasia for several reasons. The first case shows a very unusual morphological feature, the presence of several foci of squamous metaplasia in the context of a clear mesothelial proliferation. The diagnosis of epithelioid mesothelioma was achieved as a result of extensive multidisciplinary integration. Indeed, the patient’s history was negative for previous or concomitant neoplasms, nor did the radiological examinations show any other masses in the lungs or other organs. Furthermore, the loss of nuclear expression of BAP1 is a very rare event in squamous cell carcinoma [11], corroborating the hypothesis that the neoplastic areas with squamous morphology represent a metaplastic process rather than a pure squamous carcinoma. To the best of our knowledge, there is only one other report in the English-language literature [12] that describes the presence of this morphological feature, which should be recognized as it represents a potential and insidious pitfall. 

The second case shows an extremely rare variant of mesothelioma, the localized subtype. This was previously reported by our group as Clinical Picture in a peer-reviewed journal [13]. The LMM shares the same morphological, immunophenotypic, and ultrastructural characteristics of diffuse forms and is similarly classified into epithelioid, biphasic, and sarcomatoid [14]. Unlike classic mesothelioma, the LMM grows in the form of a nodule or mass, also pedunculated, without diffuse growth. For these reasons, its diagnosis is multidisciplinary as it requires the exclusion of other pleural nodules, thickening, or neoplastic pleural effusion. Neither the causal link with exposure to asbestos nor the prognosis of this neoplasm is well known. Indeed, approximately 60% of the reported patients did not have a documented history of asbestos exposure, as in our case. The largest series of cases collected describe median survival ranging from 29 to 134 months [15].

Given the uniqueness of these cases, the ultrastructural analysis provided further support in confirming the mesothelial origin of the neoplastic cells. Instead, the third case is emblematic of the difficulties that can occur in determining the histotype of a pleural tumour on a small biopsy in a patient with other neoplasms. The clinical history and the absence of exposure to asbestos suggested a pleural recurrence of breast cancer even if other immunohistochemical features, such as GCDFP-15 and BAP1 negativity, were in favour of an alternative diagnosis. TEM confirmed the mesothelioma hypothesis, showing elongated microvilli typical of mesothelial cells. Although breast cancer cells may display microvillar protrusions, they are shorter and unbranched [16]. Furthermore, another rare but well-documented risk factor for the development of mesothelioma is represented by therapeutic radiation for other malignancies [17]. Mesothelioma of the third case affects the right pleural cavity in the same site of the radiotherapy for breast cancer, suggesting a possible link between radiations and neoplastic transformation.

## 4. Conclusions

In summary, we presented this anecdotic case series to illustrate the contributory value of ultrastructural analysis in principal settings either when immunohistochemistry is inconclusive or when there is a very rare case. Electron microscopy combined with laboratory experience obtains optimal and reliable results from processing formalin-fixed material retrieved from paraffin-embedded blocks.

## Data Availability

Not applicable.
